# Ruxolitinib-dependent reduction of seizure load and duration is accompanied by spatial memory improvement in the rat pilocarpine model of temporal lobe epilepsy

**DOI:** 10.1016/j.neurot.2024.e00506

**Published:** 2024-12-05

**Authors:** Andrew Carrel, Eleonora Napoli, Kathryn Hixson, Jessica Carlsen, Yasmin Cruz Del Angel, Dana Strode, Nicolas Busquet, Vijay Kumar, Michael F. Wempe, Shelley J. Russek, Amy R. Brooks-Kayal

**Affiliations:** a Department of Pediatrics, University of Colorado School of Medicine, Aurora, CO, USA; b Department of Neurology, University of California Davis School of Medicine, Sacramento, CA, USA; c Graduate Program for Neuroscience, Center for Systems Neuroscience, Boston University, Boston, MA, USA; d Department of Neurology, University of Colorado School of Medicine, Aurora, CO, USA; e Department of Pharmaceutical Sciences, Skaggs School of Pharmacy and Pharmaceutical Sciences, University of Colorado Anschutz, Aurora, CO, USA; f Department of Chemistry, Kentucky State University, Frankfort, KY, USA; g Department of Pharmacology and Experimental Therapeutics, Boston University School of Medicine, Boston, MA, USA

**Keywords:** Epilepsy, Hippocampus, Memory, Pilocarpine rat model, Ruxolitinib, Seizure

## Abstract

Molecules with optimized pharmacokinetic properties selectively aimed at the inhibition of STAT3 phosphorylation in brain have recently emerged as potential disease modifying therapies for epilepsy. In the current study, pharmacological inhibition of JAK1/2 with the orally available, FDA-approved drug ruxolitinib, produced nearly complete inhibition of hippocampal STAT3 phosphorylation, and reduced the expression of its downstream target Cyclin D1, when administered to rats 30 ​min and 3 ​h after onset of pilocarpine-induced status epilepticus (SE). This effect was accompanied by significantly shorter seizure duration and lower overall seizure frequency throughout the 4 weeks of EEG recording, but did not completely prevent the development of epilepsy in ruxolitinib-treated male rats. Compared to DMSO-treated animals, administration of ruxolitinib also improved memory (Y maze) but did not impact motor function (open field) following SE. Taken together with our previous findings, the results of this study provide further evidence that inhibition of the JAK/STAT pathway may be a promising disease modifying strategy to reduce severity of acquired epilepsy after brain injury, but also point to the need to better understand and optimize inhibitors of this pathway.

## Introduction

According to recent data from the World Health Organization [1], epilepsy affects 50 million people worldwide, making it one of the most common neurological disorders, with an estimated 1 in 26 people developing epilepsy in their lifetime [2]. Despite the introduction of over one dozen new antiseizure medications in the past decade, the percentage of patients with treatment-resistant epilepsy remains unchanged [3]. Currently, over 35 ​% of people with epilepsy do not achieve seizure freedom [3], partly because none of the commercially available antiseizure medications target the neuropathological processes underlying epileptogenesis [3]. To make matters worse, comorbidities like cognitive disabilities and neuropsychiatric conditions impact over 1/3 of people with epilepsy [4], substantially impair their quality of life and are exacerbated by a number of antiseizure medications [5,6].

The most common form of acquired epilepsy after brain injury is temporal lobe epilepsy (TLE), characterized by recurrent, spontaneous seizures that originate from a focus in the mesial temporal region [[7], [8], [9], [10], [11], [12], [13], [14], [15]]. Evidence from our labs and others have demonstrated that in rodent models of brain injury leading to epilepsy, there is a substantial engagement of the Janus Kinase and Signal Transducer and Activator of Transcription (JAK/STAT) pathway, a signaling cascade with a prominent role in systemic inflammation and cancer. CNS dysregulation of the JAK/STAT pathway has been linked to brain inflammation and neuronal/glial survival and is a critical player in the mediation of brain insult [[16], [17], [18], [19]]. As such, its involvement has been suggested in brain disorders including epilepsy, brain cancer, TBI, ischemia and neurodegenerative disorders like Alzheimer's disease [20].

In particular, the JAK/STAT pathway has been identified as a major regulator of GABA_A_ receptor gene (*Gabr*) expression [16], as well as in the expression of genes involved in inflammation, neuronal proliferation, cell survival and gliogenesis [[21], [22], [23], [24], [25], [26], [27]], all of which are essential for normal brain function, and potentially important contributors to epileptogenesis. Furthermore, it has been established that STAT3 signaling is involved in the induction of NMDA-receptor dependent long-term depression in the hippocampus [28], a cellular mechanism critical in learning and memory [29].

Recently, studies from our groups have shown that genetic- [30] or pharmacologic-mediated [31] inhibition of STAT3 at the time of brain injury induced by status epilepticus (SE), reduces the severity of subsequent epilepsy in rodent models.

Relevant to the current study, the identification of molecules with optimized pharmacokinetics selectively aimed at the inhibition of STAT3 phosphorylation in brain may lead to the development of more effective disease modifying therapies which will inhibit seizure onset and progression, rather than merely treat the symptoms associated with the disease. In the rat pilocarpine (PILO) model, we previously showed that intraperitoneal administration of a STAT3 inhibitor (WP1066) at the time of SE produced a partial and transient inhibition of hippocampal STAT3 phosphorylation at the Tyr residue 705 that was associated with a significant reduction in the frequency of subsequent spontaneous seizures [31] but did not fully prevent seizure development. We hypothesized that the incomplete therapeutic effect might be a result of the short half-life (<30 ​min) and poor blood-brain barrier (BBB) permeability of WP1066 that resulted in only partial and transient pSTAT3 inhibition [31].

Ruxolitinib (hereinafter abbreviated as RUXO) is a newer orally bioavailable JAK/STAT signaling inhibitor. This molecule belongs to the kinase inhibitors family and is currently in use for the treatment of certain myeloproliferative neoplasms [32,33]. In the current study, using the same PILO rat model previously used for the investigation of the effects of WP1066, we sought to clarify whether RUXO administration could result in a more complete and prolonged pSTAT3 inhibition at the time of SE, and if this would translate into a more robust reduction of spontaneous seizures.

Finally, since STAT3 has been indicated as a potential regulator of (i) long-term depression [28], (ii) vestibular motor recovery after traumatic brain injury (TBI) [34], and (iii) motor learning and memory in the intrahippocampal kainic acid (IHKA) mouse model of TLE [30], we also investigated the effect of RUXO-mediated STAT3 inhibition on memory and motor function following SE.

## Materials and Methods

### Chemicals

Unless otherwise indicated, all chemicals were of analytical grade or higher and purchased from Sigma-Aldrich (St. Louis, MO). Diazepam was from Hospira (Lake Forest, IL), Ruxolitinib was synthesized by the Medical Chemistry Core at the University of Colorado School of Pharmacy (Aurora, CO).

### Animals

Adult (8 weeks old) male Sprague Dawley rats (Charles River, Wilmington, MA) were group-housed until SE (then housed individually) in a temperature-controlled vivarium with food and water *ad libitum* on a 14/10-h light/dark cycle. All experimental procedures were conducted in accordance with the National Institutes of Health's “Principles of Laboratory Animal Care” and in accordance with University of Colorado Anschutz Medical Campus Institutional Animal Care and Use Committee regulations and under approved protocols (# 00042).

### Pilocarpine-induced SE and RUXO administration

Rats were first administered 1.0 ​mg/kg methylscopolamine via intraperitoneal (i.p.) injection to block peripheral cholinergic effects. Subsequently, SE was induced in a subset of animals via i.p. administration of 385 ​mg/kg pilocarpine hydrochloride, while Sham rats received a sub-convulsive dose of pilocarpine (38.5 ​mg/kg). To reduce mortality, SE-induced rats received 6.0 ​mg/kg diazepam i.p. 1 hr after SE onset (defined as the first class 5 motor seizure), and then 3 ​mg/kg every 2 ​h in case of persistent seizures. Sham animals received 1:10 of the dose of diazepam (0.6 ​mg/kg). All rats in the RUXO group were injected with a RUXO dose of 20 ​mg/kg (the lowest dose shown to effectively inhibit STAT3 phosphorylation) dissolved in DMSO i.p. 30 min post-SE onset. A subset of animals that underwent testing beyond 3 ​h from SE onset were injected with a second RUXO dose of 10 ​mg/kg (lowest dose to produce an additive effect on STAT3 phosphorylation levels at 6 ​h) injected 150 ​min later (=3 ​h after SE onset). Vehicle controls received equivalent volumes of DMSO i.p. at the same timepoints.

### Bioanalytical pharmacokinetic analysis

#### Sample preparation and extraction

Animals were anesthetized using isoflurane and decapitated. At various time-points following RUXO administration, ∼1 ​mL whole blood was collected immediately after decapitation via aspiration from the jugular vein and placed into ice-cold EDTA-coated tubes (Becton Dickinson and Company, Franklin Lakes, NJ). Blood samples were then centrifuged at 14,000 ​rpm for 10 ​min at 4 ​°C and plasma (200 ​μL) transferred onto dry ice. In parallel, liver, kidneys and brain were harvested, weighed, and flash frozen on dry ice. All samples were stored at −80 ​°C ​± ​10 ​°C until BAPK (bioanalytical pharmacokinetic) analyses. Right before analysis, blood and other tissues were allowed to thaw on ice. The extraction solution was freshly prepared by mixing methanol:acetonitrile (1:1); stock DMSO internal standard (tofacitinib) was then spiked into the extraction solution and mixed. Vortex mixed plasma samples were added with extraction solution (250 ​μL containing internal standard), vortex mixed (5 ​s), sonicated in a water bath (5 ​min), vortex mixed (5 ​s) a second time, and then centrifuged (10,000 ​rpm for 10 ​min) using an Eppendorf mini-spin centrifuge (Hamburg, Germany). Supernatants were immediately transferred into individual wells of a 96-well plate, placed into the LEAP auto-sampler cool-stack (8.0 ​± ​1.0 ​°C) and immediately analyzed via LC/MS-MS.

Tissues thawed on ice were homogenized for 2 ​min at a 1:2 ratio (w/v) in PBS (Phosphate Buffer Solution, pH 7.4) and subsequently extracted in a 1:2 sample:extraction solution (v/v) as described for plasma.

#### RUXO standard curves

Control tissue was procured from Bioreclamation LLC [rat plasma (K2 EDTA, Lot # RAT166627); rat blood (K2 EDTA, Lot # RAT204099); rat liver (Lot # RAT205185); rat kidney (lot # RAT205190); and rat brain (Lot # RAT205198)]. Control tissues and test tissues (e.g. brain, liver, kidney) were homogenized using two volumes ice cold phosphate buffer (100 ​mM, pH 7.4; 1.0 ​g tissue per 2.0 ​mL PBS). Standard Curve samples were prepared by adding homogenate (475 ​μL) and spiked with aqueous drug solutions (25 ​μL), capped, vortex mixed and frozen (−80 ​°C). Frozen standard curve samples were removed, allowed to thaw, and extracted on a 1 ​vol (homogenate) to 2 ​vol extraction solution spiked with internal standard. Samples were mixed with extraction solution, vortex mixed, allowed to sit at room temperature (10 ​min), vortex mixed a 2nd time and centrifuged (10,000 ​rpm ​× ​5 ​min). The supernatants were then transferred to a 96-well plate and analyzed by LC/MS-MS. For plasma, blood, and tissue homogenate (kidney, cortex and liver), nine-point standard curves (n ​= ​4 ​± ​SD) representing concentrations between 1 and 1200 ​ng/mL were prepared. All standard curve data were fitted to a 1/x^2^ weighted linear regression; these standard curves were used to determine apparent drug concentrations from the extracted samples.

#### LC/MS-MS

Mass spec (LC/MS-MS) analysis was performed with the use of the Sciex API 4000 System (Applied Biosystems; Foster City, CA) equipped with a Shimadzu HPLC (Shimadzu Scientific Instruments, Inc.; Columbia,MD) and Leap auto-sampler (LEAP Technologies; Carrboro, NC). Liquid chromatography employed an Agilent Technologies, Zorbax extended-C18 50 ​× ​4.6 ​mm and 5-μm column; a column guard was used at 40 ​°C with a flowrate of 0.4 ​mL/min. The mobile phase consisted of A: HPLC H_2_O, 10 ​mM ammonium acetate, 0.1 ​% formic acid and B: 1:1 methanol:acetonitrile. Between samples the auto-sampler was washed with a 1:1:1:1 mixture of ACN:MeOH:IPA:water. The chromatography method used was 95 ​% A for 1 ​min; ramped to 95 ​% B at 4.5 ​min and held for 4 ​min; next, the solvent was brought back to 95 ​% A at 10 ​min and held for 2 ​min (12 ​min total run time). The mass spectrometry conditions used were as previously described [31] but with slight modifications: 1) Curtain gas of 10 (CUR; arbitrary setting) and collisionally activated dissociation (CAD; set at 12) gas were nitrogen; 2) ion source gas one (GS1) and two (GS2) set at 30; 3) ion-spray voltage of 5500 ​V; 4) temperature of 450 ​°C; 5) Q1 and Q3 set to Unit resolution with a dwell time of 200 ​ms; 6) declustering potential (DP), collision energy (CE), and collision cell exit potential (CXP) are voltages (V). The internal standard used was tofacitinib (IS) which had a retention time of 4.1 ​min; MS/MS: 313.1 ​*m*/*z* → 149.2 ​*m*/*z*; DP ​= ​76, CE ​= ​41, CXP ​= ​12; and RUXO had a retention time of 5.1 ​min, 307.1 → 186.0 ​*m*/*z*; DP ​= ​61, CE ​= ​35, CXP ​= ​10.

### EEG acquisition and analyses

To accurately analyze electrographic seizure frequency, two bilateral subdural stainless-steel screws (4.0 ​mm posterior, 2.5 ​mm lateral relative to the bregma) were placed over the temporolimbic cortices as previously described [31]. Additional stainless-steel screws were placed on each side of the brain behind lambda (*i.e.*, over the cerebellum) and were used as reference and ground electrodes. Animals were allowed to recover from surgery for 1 week before proceeding with any further experimentation. Epileptic rats were video-EEG monitored 24 ​h/day for 4 weeks using Pinnacle digital video-EEG systems [31].

Off-line data analyses were performed by technicians blinded to all experimental parameters to both identify electrographic seizures and analyze indices of electrographic SE after RUXO (or vehicle) treatment. Electrographic seizures analysis was performed as described in detail elsewhere [31]. SE power analysis was conducted using a Fast Fourier Transformation (FFT) in LabChart v7.3.7 software from ADInstruments (Colorado Springs, CO). Similar to our prior studies on WP1066 [31], for the initial analyses, a rectangular window was used to determine maximum power at SE onset, as well as total power from SE onset until reaching 25 ​% of maximum power (defined as the end of SE). Total power metric hence incorporates both SE severity and duration. For the short-term analyses, the total power in each frequency band (1–4 ​Hz, 4.1–8 ​Hz, 8.1–13 ​Hz, 13.1–30 ​Hz and 30.1–100 ​Hz) was averaged across both EEG data channels. Motor seizures were scored utilizing standard behavioral classification [35].

### Behavioral testing

In order to identify potential RUXO-dependent behavioral effects, four weeks after SE induction rats underwent a battery of tests designed to evaluate locomotion and cognition.

#### Open field

This test is widely used to measure exploratory behavior, locomotion, hyperactivity, and some aspects of anxiety-related behavior in rodent models [36,37], leveraging the conflicting innate tendencies of rodents to avoid open spaces and to explore novel environments. The testing apparatus consisted of a 100 ​× ​100 ​cm wall-enclosed arena with 40 ​cm high walls. Rats were monitored for 10 ​min, and the total distance traveled (cm) and time spent in the center zone were recorded using a video tracking system (Noldus Ethovision XT).

#### Spontaneous alternation behavior

The Spontaneous Alternation Behavior (SAB) test is routinely used to assess hippocampal function [38] and employed in pharmacological studies of spatial and reference memory retention in rodents [39]. Briefly, rats were introduced into a symmetrical Y-maze (3 arms 10 ​cm wide and 45 ​cm deep, angled at 120°), and allowed to freely explore the new environment. The ratio of correct alternations was calculated over the course of 22 arm entries (20 alternations) or 8 ​min, whichever came first. Animals with deficits in spatial cognition and working memory were expected to explore the Y maze more randomly than control animals [39].

### RT-PCR

RNA was extracted from microdissected hippocampal DG tissue with the use of TRIzol reagent (Invitrogen, Carlsbad, CA). For cDNA synthesis, 0.500 ​μg of RNA was processed with the SuperScript II reverse transcription kit (Invitrogen) following manufacturer's specifications, and then diluted 1:4 for storage and subsequent RT-PCR. For RT-PCR reactions, each sample was run in triplicate and each 25-μL reaction contained 1.25 ​μL Taqman primer and probe for ICER (CREM Rn00569145_m1) or Cyclophilin A (housekeeping gene; PPIA Rn00690933_m1), 12.5 ​μl of Taqman Master mix, 6.25 ​μl of DEPC water (all from Applied Biosystem) and 5.0 ​μl of complementary DNA. RT-PCR was performed on the SDS-7500 PCR machine (Applied Biosystems). The RT-PCR conditions were: 1 cycle of 50 ​°C for 2 ​min, 1 cycle of 95 ​°C for 10 ​min, and 40 cycles of 95 ​°C for 15 ​s and 60 ​°C for 1 ​min.

### Western blotting

Western blot analysis was performed with modifications of published protocols [31,40]. Whole hippocampus or microdissected DG, CA1 and CA3 subregions were sonicated (Branson Digital Sonifier set at 30 ​% power) for 10–12 ​s in 1x RIPA buffer (added with 1x Protease and Phosphatase inhibitors). Lysates were incubated on a rotator at 4 ​°C for 15 ​min, then cleared at 13,000 RPM for 10 ​min. Extracted protein (20–30 ​μg) were loaded onto 8–10 ​% SDS-polyacrylamide gels, run for ​∼ ​1.5 ​h at 115 ​V, transferred onto nitrocellulose membranes (33V for 2 ​h) and blocked in 5 ​% milk/trisphosphate-buffered saline (pH 7.6) with 0.05 ​% Tween-20 (TBS-T) for 1 ​h at RT. Subsequently, membranes were incubated overnight at 4 ​°C in 5 ​% bovine serum albumin/TBS-T with primary antibodies raised against the following targets: Cyclin D1 [Cell Signaling Technologies (CST) # #2978; 1:500]; STAT3 phosphorylated at Tyr705 (anti-pSTAT3; CST #9131S; 1:1000) and total STAT3 (CST #4904S; 1:6000); JAK2 phosphorylated at Tyr1007/1008 (anti-pJAK2; CST; 1:400) and total JAK2 (1:2000); ICER (Millipore/Sigma #SAB2500271; 1:100). Membranes were then washed in TBS-T and incubated with anti-rabbit (GE Health Care 1:5000) or anti-mouse (Jackson Immunoresearch Laboratories; 1:25,000) conjugated to horseradish peroxidase (HRP) secondary antibodies for 1 ​h in 1 ​% milk/TBS-T. Immunoreactive bands were visualized using Super Signal West Pico chemiluminescent substrate (Pierce). Beta-actin (1:60,000 dilution; Millipore/Sigma) was used as the loading control. Bands were quantified using Image J (v. 1.42q).

### Immunofluorescence and imaging

Four weeks after SE rats were anesthetized and perfused transcardially with cold PBS followed by 4 ​% paraformaldehyde (PFA) in 0.1 ​M phosphate buffer. Whole brains were extracted, postfixed overnight in 4 ​% PFA, and cryoprotected in 30 ​% sucrose. Cryoprotected brains were embedded in OCT (Tissue-Tek, Sakura Finetek, Torrance, CA) and serially sectioned in the coronal plane. To detect degenerating neurons, 10 mounted brain sections (14 ​μm) from a 1-in-20 series (−2.3 ​mm to −4.8 ​mm from the bregma) from each brain (n ​= ​3 animals per group) were stained with the anionic fluorochrome Fluoro-Jade B (FJB, Histochem Inc., Jefferson, AR) as described [31]. Images were obtained with a Nikon Eclipse TE2000-U fluorescence microscope at 10x magnification. FJB-positive neurons were counted in the CA1, CA3, and hilar region of the hippocampus using ImageJ. Cell counts were performed by an investigator blinded to treatment group.

For the assessment of mossy fiber sprouting (MFS), rats were perfused and brains fixed as described above, and brain sections were stained using an anti-Zinc Transporter 3 (ZnT3) antibody [30,41]. Briefly, mounted slides (14 ​μm) were thawed at RT, washed 1 ​× ​5 min in PBS, 2 ​× ​5 min in 0.3 ​% Triton/PBS (T-PBS), and then blocked for 90 ​min in blocking buffer (0.3 ​% Triton ​+ ​10 ​% normal goat serum in PBS). Slides were incubated overnight in rabbit anti-ZnT3 primary antibody (1:500 dil; Synaptic Systems #197 002) in blocking buffer. The following day slides were washed 3 ​× ​5min in T-PBS and subsequently incubated for 1 ​h at RT in AlexaFluor 568 goat anti-rabbit secondary antibody (1:700 dilution in blocking buffer; Invitrogen #A11011). Slides were then washed 1 ​× ​5 min in T-PBS and 3 ​× ​5min in PBS. Images were obtained of the upper and lower DG for each section using a Nikon Eclipse TE2000-U fluorescence microscope and analyzed with ImageJ.

### Statistical analysis

Statistical analysis was performed with the software GraphPad Prism, version 10.1.2. Wherever indicated, outliers were identified with the ROUT method [Q ​= ​1 ​% [42]] and excluded from statistical analysis. Sham controls are shown in each figure as reference, but were not included in the statistical analysis. Differences between Sham and epileptic rats for most individual outcomes reported in the current study have already been shown elsewhere [31]. Normality was assessed with the Shapiro-Wilk test. Non-parametric analysis was performed with data that did not follow a Gaussian distribution.

To account for time and treatment effects, seizure duration data have been analyzed by 2-way ANOVA followed by Tukey's post-hoc test, since they followed a Gaussian distribution. Conversely, total number of seizures and seizure severity data did not pass the normality test; as such between-group differences (DMSO vs RUXO) have been analyzed with the Mann-Whitney test.

Differences in phosphorylation levels of STAT3 and JAK2 at different time points were analyzed either using parametric (Unpaired t) or non-parametric (Mann-Whitney) tests, depending on the distribution of the data. Other statistical tests used for individual comparisons are described in the correspondent figure legends.

## Results

### RUXO pharmacokinetics

**Fig. 1:**
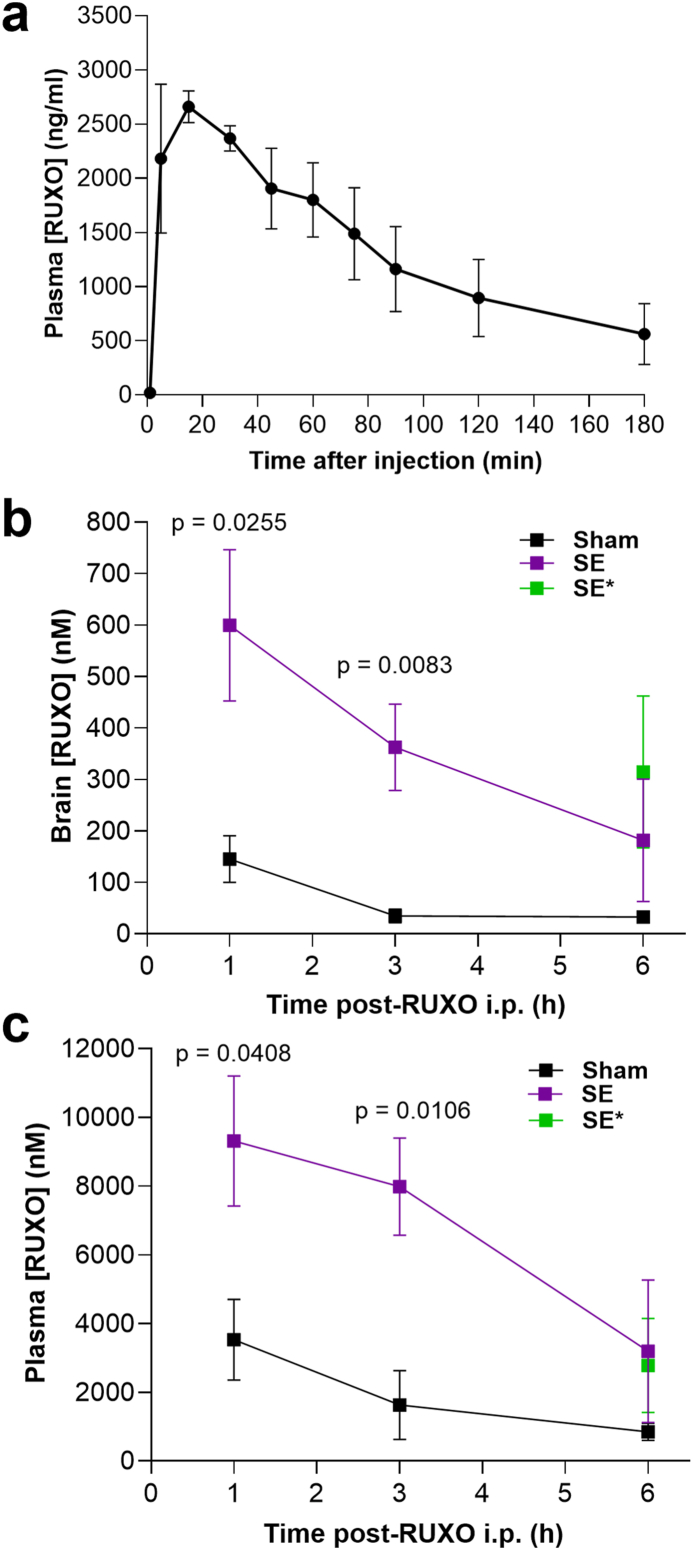
**RUXO pharmacokinetics.** Concentration of RUXO was assessed in plasma isolated from tail blood collected at 1, 5, 15, 30, 45, 60, 75, 90, 120 and 180 ​min after ip injection of 20 ​mg/kg of RUXO (**a**). Data are shown as mean ​± ​SEM (n ​= ​4). RUXO brain (**b**) and plasma (**c**) concentration were assessed at 1, 3 and 6h post-SE in SE and Sham treated with 20 ​mg/kg RUXO at 30 ​min after SE onset. In addition a separate group of animals was also treated with RUXO at 10 ​mg/kg 3 ​h after SE-onset and plasma and brain concentration assessed at 6 ​h (shown in green and labeled SE∗). Statistically significant differences were noted between Sham and SE at 1 ​h and 3 ​h by Unpaired *t*-test. P values are shown in panels b and c. No differences were recorded between groups at 6 ​h by Mann-Whitney test.

Based on the reported *in vitro* RUXO IC_50_ [27,43], rats (n ​= ​4 per group per time point) were dosed i.p. with 20 ​mg/kg RUXO and blood was collected via tail-bleed at 1, 5, 15, 30, 45, 60, 90, 120, and 180 ​min (Fig. 1a). The *in vivo* RUXO half-life in blood was calculated at 75–90 ​min. In order to determine RUXO brain bioavailability, we collected brain and blood samples of SE and Sham animals at 1, 3, and 6 ​h post-administration. Brain samples consisted of cortical and subcortical structures (no cerebellum or medulla) after removal of right and left hippocampi. The mean brain RUXO concentration (mean ​± ​SD) in Sham was 145.0 (±90.9) nM at 1 ​h, 34.8 (±26.6) nM at 3 ​h and 32.8 (±8.5) nM at 6 ​h post-injection (Fig. 1b). In SE animals, calculated RUXO brain concentrations were 599.8 (±294.3), 362.5 (±167.5), and 181.8 (±266.2) nM at 1, 3, and 6 ​h, respectively, with statistically significant differences recorded at 1 and 3 ​h compared to Sham (Fig. 1b). The 4- to 10-fold increase in brain RUXO concentrations observed in SE animals at all time points are in agreement with findings of seizure-induced disruption of BBB integrity within the first hour of SE [[44], [45], [46], [47]], leading to increased BBB permeability and access of larger molecules. Brain concentrations in a subset of SE animals (n ​= ​4) administered a supplemental 10 ​mg/kg dose of RUXO 3 ​h after SE onset was 417.8 (±293.7) nM at 6 ​h (Fig. 1b, in green).

In plasma, RUXO mean concentrations at 1, 3, and 6 ​h were respectively 3531 (±2351), 1630 (±2006), and 845.8 (±506) nM in controls, vs 9317 (±3785), 7984 (±2839), and 3792 (±4635) nM in SE animals (Fig. 1c). Plasma concentrations at 6 ​h in SE rats (n ​= ​4) given the supplemental 10 ​mg/kg dose at 3 ​h post-SE had a mean concentration of 2781 (±2372) nM (Fig. 1c, in green).

### RUXO substantially attenuates SE-dependent increased phosphorylation of STAT3

**Fig. 2:**
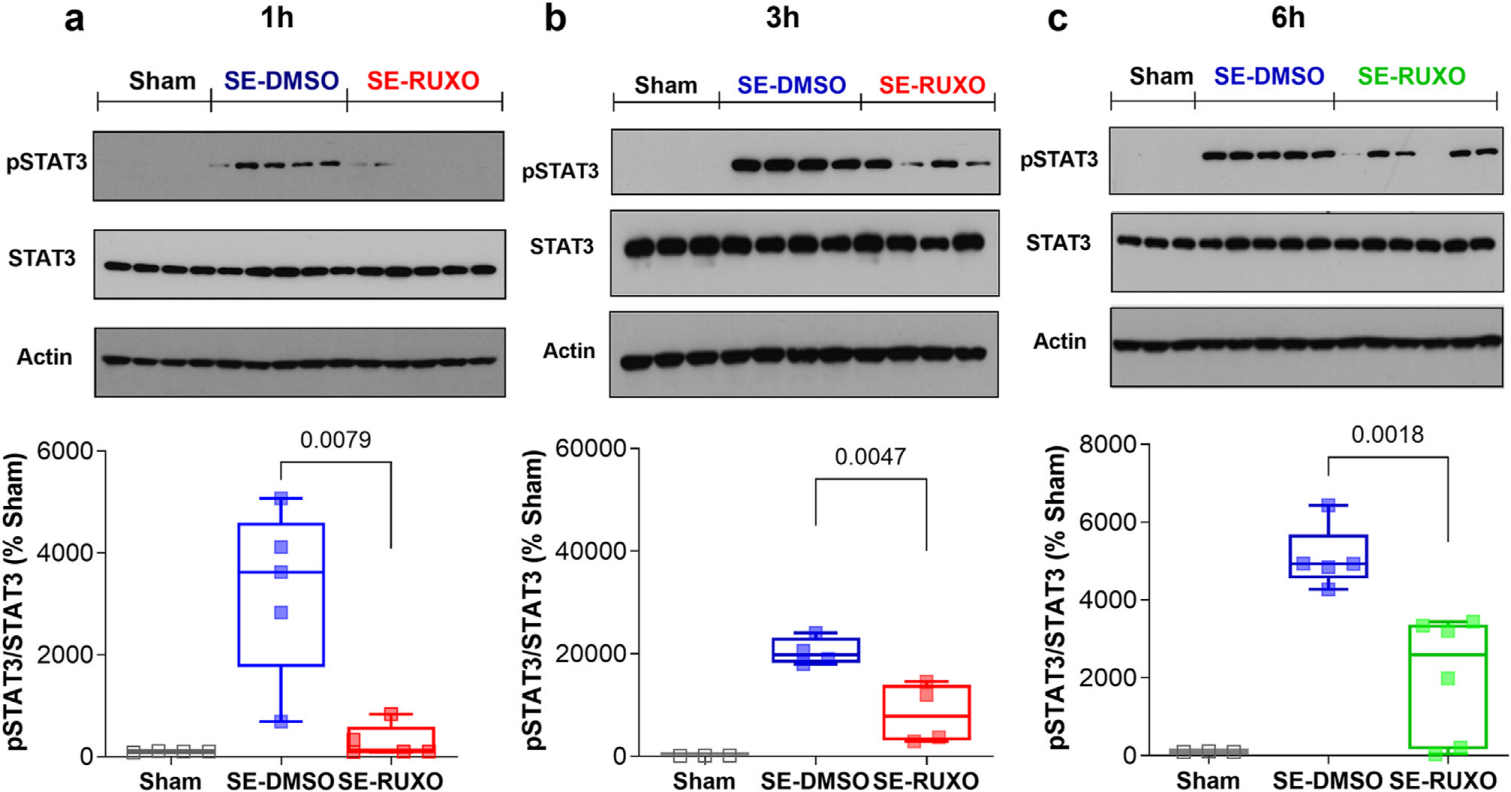
**RUXO-mediated sustained inhibition of STAT3 phosphorylation in hippocampus after induction of SE.** Representative western blot and bar graphs showing STAT3 phosphorylation levels at Tyr 705 in whole hippocampus at 1 ​h (**a**; Sham n ​= ​4, SE-DMSO n ​= ​5, SE-RUXO n ​= ​5), 3 ​h ​(**b**; Sham n ​= ​3; SE-DMSO n ​= ​4, SE-RUXO n ​= ​4) and 6 ​h (**c**; Sham n ​= ​3; SE ​+ ​DMSO n ​= ​5; SE ​+ ​RUXO n ​= ​6) after pilocarpine injection. SE-induced animals received a 20 ​mg/kg dose of RUXO (or equivalent volume of DMSO) 30′ after induction of SE. Blue squares and and outlined bars indicate vehicle-treated SE-animals; red squares and outlined bars indicate RUXO-treated SE animals; green squares and outlined bar indicate SE rats receiving a second dose of RUXO (10 ​mg/kg) 3 ​h after SE. Phosphorylated STAT3 data are normalized by total STAT3 and expressed as % of Sham. Graphs show box and whiskers (median and min-max range), as well as individual values. Differences in STAT3 phosphorylation levels between DMSO- and RUXO-treated rats were analyzed by Mann-Whitney (**a**) or unpaired *t*-test (**b** and **c**). See also Supplementary Fig. 1a–c and Supplementary Fig. 2a–c showing total STAT3 and pSTAT3 levels normalized by β-actin.

Previous data from our group showed that the JAK/STAT pathway is activated following pilocarpine-mediated SE in adult rats [16,31] and that WP1066 administered at the time of SE leads to a partial and brief (<3hr) reduction of the phosphorylation levels of STAT3, accompanied by decreased severity of subsequent SRS [31]. Compared to vehicle-treated animals, 20 ​mg/kg RUXO administered at SE onset attenuated SE-mediated STAT3 phosphorylation (pSTAT3) in whole hippocampus by 11-fold at 1 ​h post-administration (Fig. 2a), a response that was sustained (though to a lesser extent; 2.5-fold) at 3 ​h (Fig. 2b). A second injection of 10 ​mg/kg RUXO 3 ​h after SE onset, led to a more sustained decrease (2.5-fold) in pSTAT3 levels, evaluated 6 ​h after the initial dose (Fig. 2c).

**Fig. 3:**
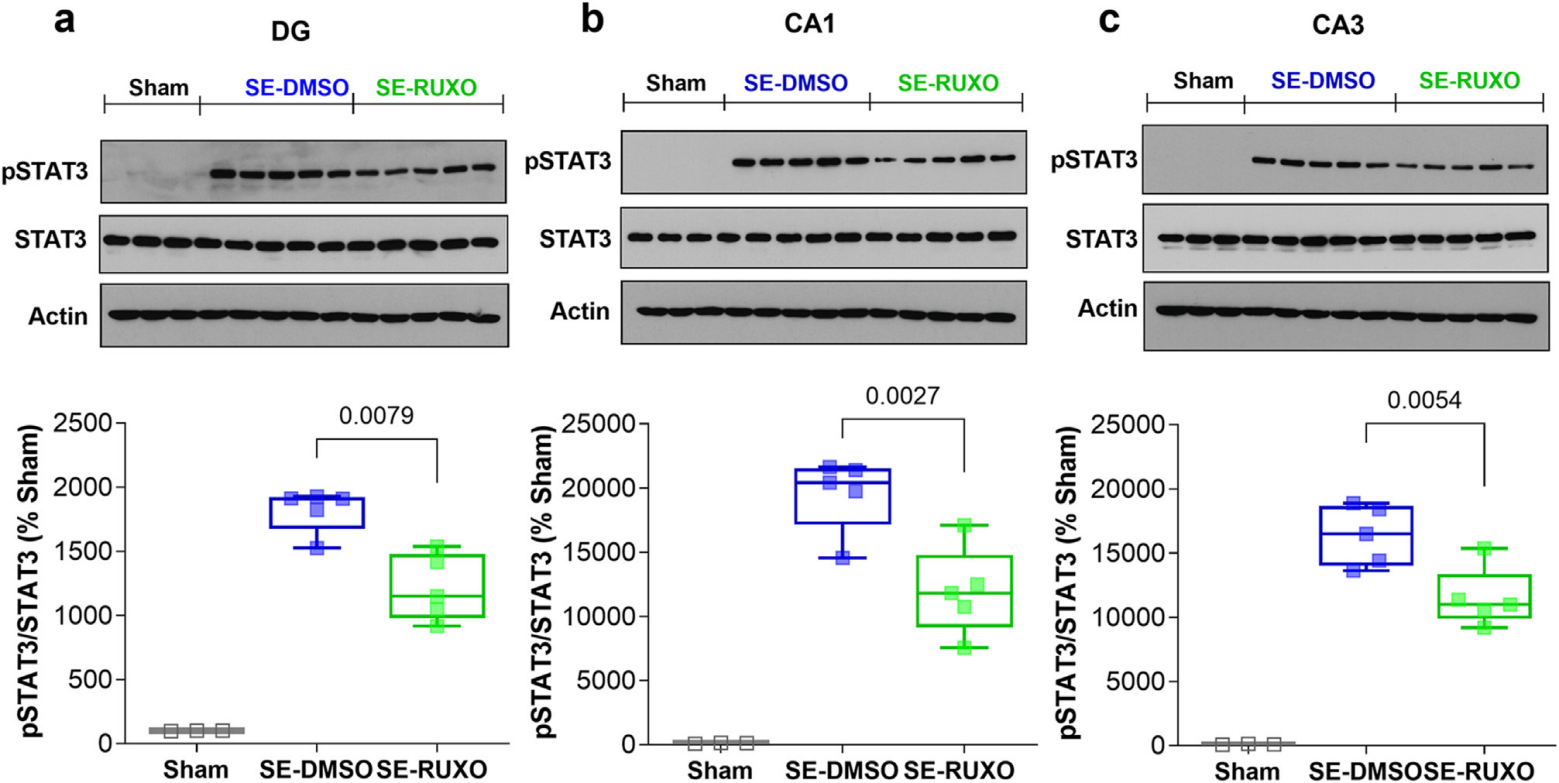
**RUXO-mediated inhibition of STAT3 phosphorylation in hippocampal subregions 6 ​h after induction of SE.** SE-mediated phosphorylation of STAT3 was assessed in DG (**a**), CA1 (**b**) and CA3 (**c**), 6 ​h after pilocarpine i.p. administration in Sham (n ​= ​3), DMSO (n ​= ​3) and RUXO (n ​= ​5) animals. Green boxes and squares indicate SE rats treated with a first dose (20 ​mg/kg) of RUXO 30 ​min after SE induction and a second dose (10 ​mg/kg) 3 ​h after SE. Data are normalized by total STAT3, expressed as % of Sham and shown as box and whiskers (min-max range). Total STAT3 levels normalized by β-actin are shown in Supplementary Fig. 1d and e. Phospho-STAT3 levels normalized by β-actin for the same subregions are also shown in Supplementary Fig. 2d and e. Statistical analysis was performed by unpaired *t*-test (**a** and **c**) or Mann-Whintey test (**b**).

To test whether the changes in pSTAT3 observed in the SE groups were driven by one particular hippocampal subregion [*i.e.*, CA1, CA3, or dentate gyrus (DG)], whole hippocampi collected at 6 ​h post-RUXO administration were microdissected and subregions from both left and right hippocampi from each animal were pooled. All three subregions showed similar patterns of activation, with SE animals (DMSO and RUXO) displaying substantially elevated levels of pSTAT3 compared to Sham (Fig. 3a–c). RUXO-treated groups had significantly lower (0.67-, 0.61-, and 0.70-fold respectively) pSTAT3 levels compared to vehicle-treated animals in DG, CA1 and CA3 (Fig. 3a–c). This is notably different from what was previously observed by our group following administration of WP1066, which resulted in no difference in STAT3 phosphorylation relative to vehicle-treated rats at 6 ​h after the onset of SE [31].

Of note, in a mouse model of traumatic brain injury (TBI), we previously showed that total STAT3 expression can increase following brain insult [34]. This led us to investigate this phenomenon in our rat model of epilepsy. We found no changes in total hippocampal STAT3 at 1 or 3 ​h post-SE or in CA1 and CA3 6 ​h after SE (Supplementary Fig. 1a and b). However, a statistically significant increase in total hippocampal STAT3 normalized to β-actin was observed 6 ​h after SE onset in both DMSO and RUXO rats (Supplementary Fig. 1c). Based on these findings, we elected to normalize pSTAT3 expression both to total STAT3 (Fig. 2, Fig. 3) and β-actin (Supplementary Fig. 2), obtaining similar results.

**Fig. 4:**
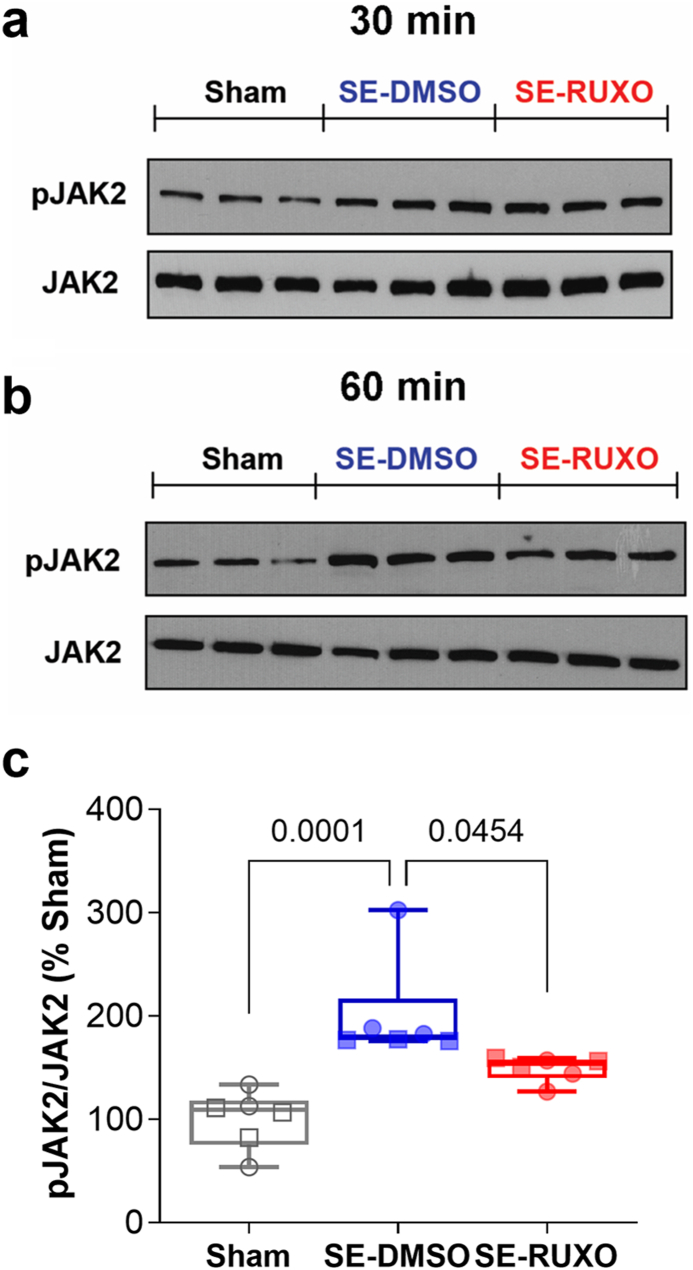
**Effect of RUXO on SE-mediated JAK2 activation.** SE-mediated increased in phosphorylation of JAK2 was assessed in hippocampus of Sham (n ​= ​3), DMSO-treated (n ​= ​3) and RUXO-treated (n ​= ​3) rats at 30 (**a**) and 60 (**b**) min after SE. Phosphorylation levels were obtained upon normalization with total JAK2. Data are expressed as % of Sham and shown as box and whiskers (min-max range). Due to the short time interval between sample collections, and the absence of intra-group significant differences recorded for pJAK2 levels at 30 and 60 ​min (see Supplementary Fig. 3), data were combined for each experimental group and statistical analysis performed by Kruskal-Wallis test followed by Dunn's post-hoc test.

It has been suggested that RUXO may reduce STAT3 activation via JAK2 inhibition [48]. Based on this premise, we tested levels of pJAK2 (the active form of JAK2, phosphorylated at Tyr1007/1008) at 30 and 60 ​min following SE onset in whole hippocampi (Fig. 4a and b). A preliminary intragroup analysis of pJAK2 levels at 30 and 60 ​min did not show any significant difference between the two time points for Sham, DMSO- and RUXO-treated rats (Fig. 4a, b; Supplementary Fig. 3). For this reason, and due to the small time window (30 ​min), data obtained for each treatment group at the two time points were pooled. Overall, and similar to what was observed for pSTAT3 at 1 and 3 ​h, expression of pJAK2 in SE rats was increased relative to Sham (2-fold; Fig. 4c), while RUXO-treated rats had pJAK2 levels that were only 0.75-fold of vehicle-treated.

### RUXO treatment impacts seizure duration and frequency

**Fig. 5:**
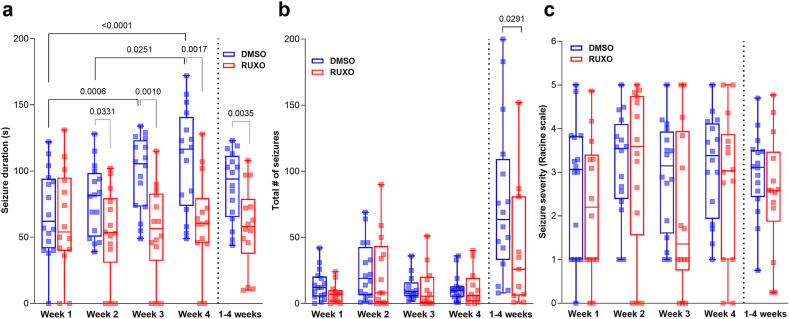
**RUXO reduces seizure duration and overall frequency, but not severity.** Seizure duration (**a**) was evaluated per week as well as overall throughout the 4 weeks of recording. For weekly intervals, statistical analysis was performed with 2-way ANOVA followed by Tukey's multiple comparisons. RUXO administration (single 20 ​mg/kg dose 30 ​min post-SE, or 20 ​mg/kg dose 30 ​min post-SE followed by a second 10 ​mg/kg dose 3 ​h after SE) has a statistically significant effect on the observed differences in seizure duration [F (1, 28) ​= ​8.535; p ​= ​0.0068] over time [F (2.917, 81.67); p ​= ​0.0007]. RUXO administration decreased the total duration of seizure in the 4 weeks of recording (unpaired *t*-test). (**b**) Seizure frequency was calculated both weekly and overall in the 4 weeks of recording in SE animals treated with vehicle (DMSO) or 20 ​mg/kg RUXO as described in the methods section. Due to the non-gaussian distribution of some of the data, differences in weekly as well as overall seizure frequency between DMSO and RUXO were evaluated with the Mann-Whitney test. Outliers, identified with the ROUT method (Q ​= ​1 ​%; see Suppl Fig. 4b), were excluded from statistical analysis. (**c**) Mean seizure severity per week as well as overall (week 1–4) was assessed using the Racine scale as described in the Methods section. Statistical analysis was performed between DMSO- and RUXO-treated groups with the Mann-Whitney test for both weekly and overall severity. All data are shown as box and whiskers, representing median and range (minimum and maximum value), as well as individual values.

To examine whether the reduction in STAT3 phosphorylation after RUXO administration altered SE severity or the development of SRS, rats implanted with subdural electrodes were continuously video-EEG recorded through SE and for the following 4 weeks. Analysis of pilocarpine-induced SE revealed that RUXO treatment did not impact any of the measured electrographic parameters of SE. No differences in total power during SE were recorded between DMSO and RUXO groups in any of the frequency domains of EEG recordings [delta (0–4 ​Hz), theta (4–8 ​Hz), alpha (8–13 ​Hz), beta/gamma (13–30 ​Hz), 30–50 ​Hz, 50–70 ​Hz, or 70–100 ​Hz bandwidths] (Supplementary Fig. 4a). As previously reported, recurrent spontaneous seizure duration increased with time after SE in vehicle-treated animals (Fig. 5a). Compared to the vehicle-treated group, RUXO-treated rats had statistically significant shorter seizures beginning at week 2, sustained at week 3 and 4 (Fig. 5a). Similarly, a statistically significant difference between the two groups was noted also when evaluating overall seizure duration across 4 weeks (Fig. 5a).

In terms of seizure load, RUXO impacted total number of seizures assessed in the 4 weeks of recording (mean ​± ​SE: 76.31 ​± ​14.70, n ​= ​16 for DMSO; 40.73 ​± ​14.35, n ​= ​11 for RUXO; p ​= ​0.0291; Fig. 5b; Supplementary Fig. 4b). Conversely, behavioral severity, scored on the Racine scale [35], was not different between the two treatment groups, either weekly or throughout the 4 weeks of recording (Fig. 5c). Further, the fraction of electrographic seizures that had an obvious behavioral correlate was not different between the RUXO and vehicle treated groups (Supplementary Fig. 4c).

### RUXO administration does not impact pilocarpine-dependent neuronal cell death or mossy fiber sprouting

**Fig. 6:**
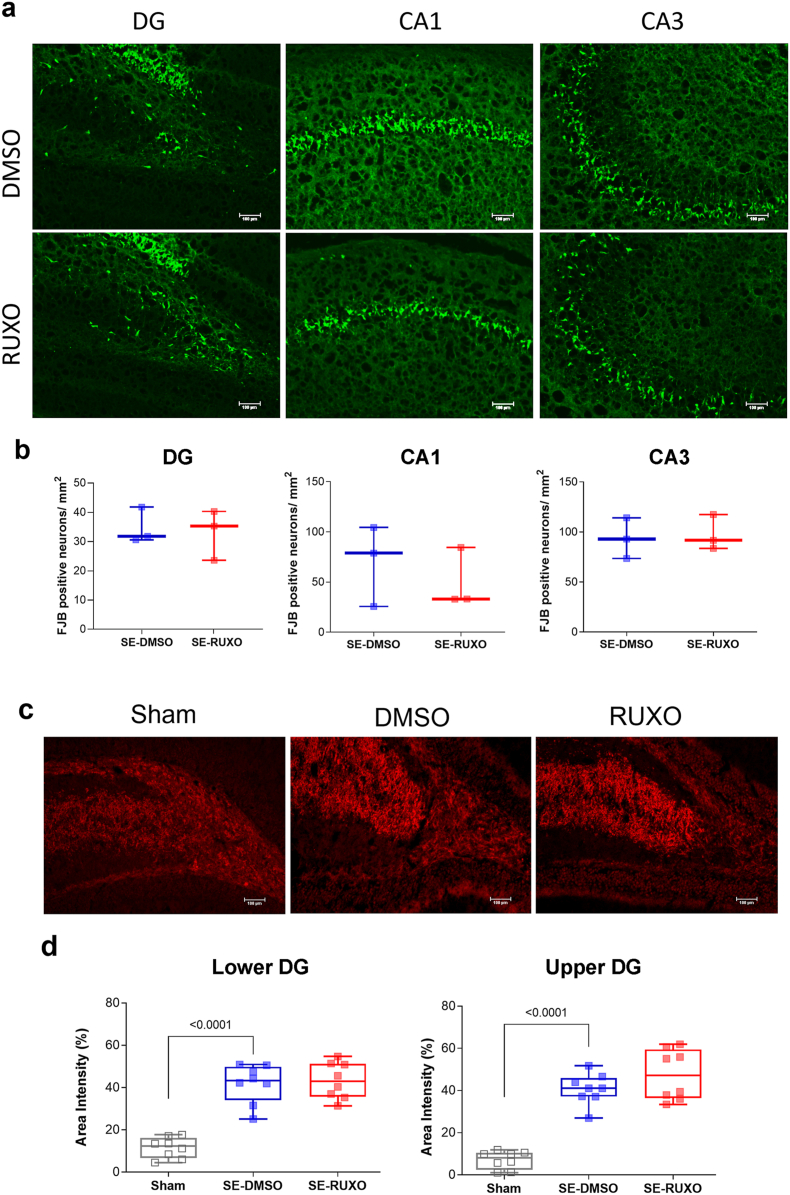
**RUXO does not protect against pilocarpine-induced acute or chronic neuronal loss nor mossy fiber sprouting.** (**a**) Representative images of Fluoro-Jade B (FJB)-stained DG, CA1 and CA3 subregions. Mounted coronal brain sections (14 ​μm) were stained with FJB to selectively stain dying neurons 48 ​h post-SE. Approximately 10 sections from a 1-in-20 series of sections from each brain (SE- RUXO, n ​= ​3; SE-DMSO, n ​= ​3) were processed. (**b**) FJB-positive cells were counted blindly within standardized areas of the DG, CA1 and CA3 regions of the hippocampus using ImageJ Analysis software (v. 1.53a). Number of FJB-positive cells in DG, CA1, or CA3, are reported as mean ​± ​SEM. Statistical analysis was performed with either unpaired Student's *t*-test (DG and CA3), or Mann-Whintey (CA1). (**c**) Representative images of ZnT3 staining in DG 4 weeks after SE induction. (**d**) ZnT3 immunoreactivity was quantified in the inner molecular layers of the upper and lower blades of the DG in 3 sections (from anterior to posterior) and values averaged. Sham animals showed no labeling in upper or lower DG while pilocarpine-injected rats showed presence of ZnT3 fluorescent staining indicative of mossy fiber sprouting (MFS). No statistically significant difference was observed in ZnT3 staining intensity between DMSO- (n ​= ​3) and RUXO-treated (n ​= ​3) rats by one-way ANOVA followed by Fisher's LSD post-hoc test. All images were taken with a Nikon Eclipse TE2000-U fluorescent microscope at 10x magnification. Fluorescence intensity was quantified with ImageJ on 16 bit-converted images.

Experiments were performed to determine whether treatment with RUXO impacted SE-induced hippocampal cell death or mossy fiber sprouting (MFS). Cell death was analyzed 48 ​h after SE onset in the hilar region of the DG, CA1, and CA3 subregions of the hippocampus using Flouro-Jade B (FJB) staining. Based on previous findings from our group [31] which showed no staining in controls, FJB staining was not performed in non-epileptic rats. FJB-positive cells were counted in each hippocampal subregion (n ​= ​3 animals/group) of DMSO- and RUXO-treated rats only. No statistically significant difference in cell death was noted between the two groups (Fig. 6a, b).

Mossy fiber sprouting in the hippocampal dentate granule cell axons is a hallmark of TLE in both animals and humans [30,49,50]. Coronal sections obtained from Sham or epileptic rats (4 weeks after SE) were stained with an antibody against the MFS marker zinc transporter 3 (ZnT3). The intensity of ZnT3 staining in DG of SE animals was significantly higher than Sham (Fig. 6c, d) but not different between vehicle and RUXO.

### RUXO administration 30 ​min and 3 ​h post-SE improves spatial memory function in epileptic rats

**Fig. 7:**
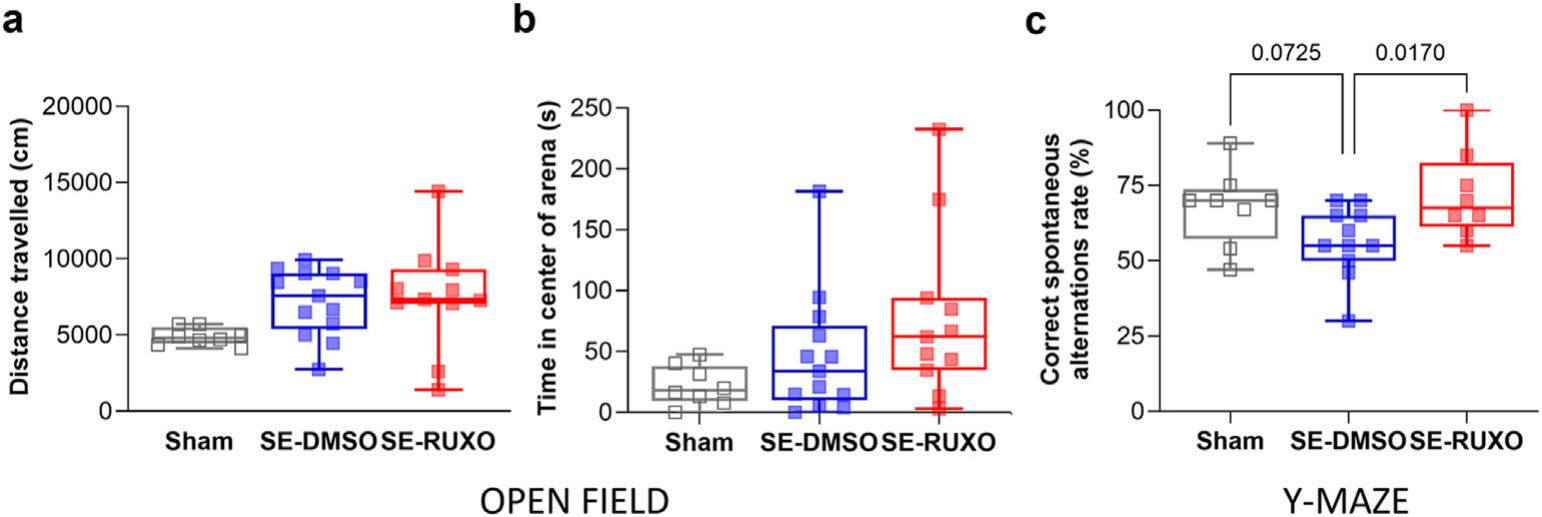
**Effect of SE induction and RUXO administration on locomotion, anxiety and spatial working memory.** (**a**) Total distance traveled (in cm) was recorded in the open field for 10 ​min in Sham (n ​= ​8), SE-DMSO (n ​= ​13) and SE-RUXO (n ​= ​11) male rats. No difference was observed in distance traveled among the 3 groups (ANOVA followed by Fisher's LSD test). (**b**) Time (s) spent in the center of arena in the open field test did not show any difference among the 3 groups (Kruskal-Wallis followed by Dunn's post-hoc test). (**c**) Rate of spontaneous alternations in Sham (n ​= ​8), SE-DMSO (n ​= ​11) and SE-RUXO (n ​= ​8) rats. Five animals were excluded from analysis due to either complete lack of movement (n ​= ​2 SE-DMSO and 2 SE-RUXO) or seizing (n ​= ​1 SE-RUXO) during test. One-way ANOVA showed a treatment effect (F ​= ​3.676, p ​= ​0.0405) and Fisher's LSD post-hoc test showed a statistically significant difference between SE-RUXO and SE-DMSO animals, while the 20 ​% difference recorded between Sham and SE-DMSO did not reach statistical significance (p ​= ​0.0725).

Distance traveled in the open field (expressed in cm) was used to test the contribution of SE and RUXO on rats’ locomotor ability (Fig. 7a). No statistically significant difference among the three groups analyzed (Sham, DMSO and RUXO) was observed in terms of total ambulatory distance. As this parameter was not different across groups, we went on to analyze the effect of RUXO on thigmotaxis, or the tendency of a subject to remain close to walls, index of anxiety-like behavior (Fig. 7b). This behavior was also not impacted by the administration of RUXO.

Y-maze testing was employed to assess spatial memory [39]. As rodents typically show a tendency to enter a less recently visited arm, entering arm C after having visited A then B, is a correct alternation pattern, whereas reentering A would indicate incorrect alternation. Sham and SE-RUXO-treated rats displayed a similar tendency for correct alternations (67.8 ​% and 71.9 ​% of the times respectively; Fig. 7c). On the other hand, vehicle-treated SE-DMSO animals performed correct alternations only in 56.5 ​% of the cases (Fig. 7c).

Taken together, these findings suggest that administration of RUXO 30 ​min post-SE onset improves spatial memory, but has no impact on locomotion or explorative behavior.

### Effect of RUXO on downstream targets of the JAK/STAT pathway

**Fig. 8:**
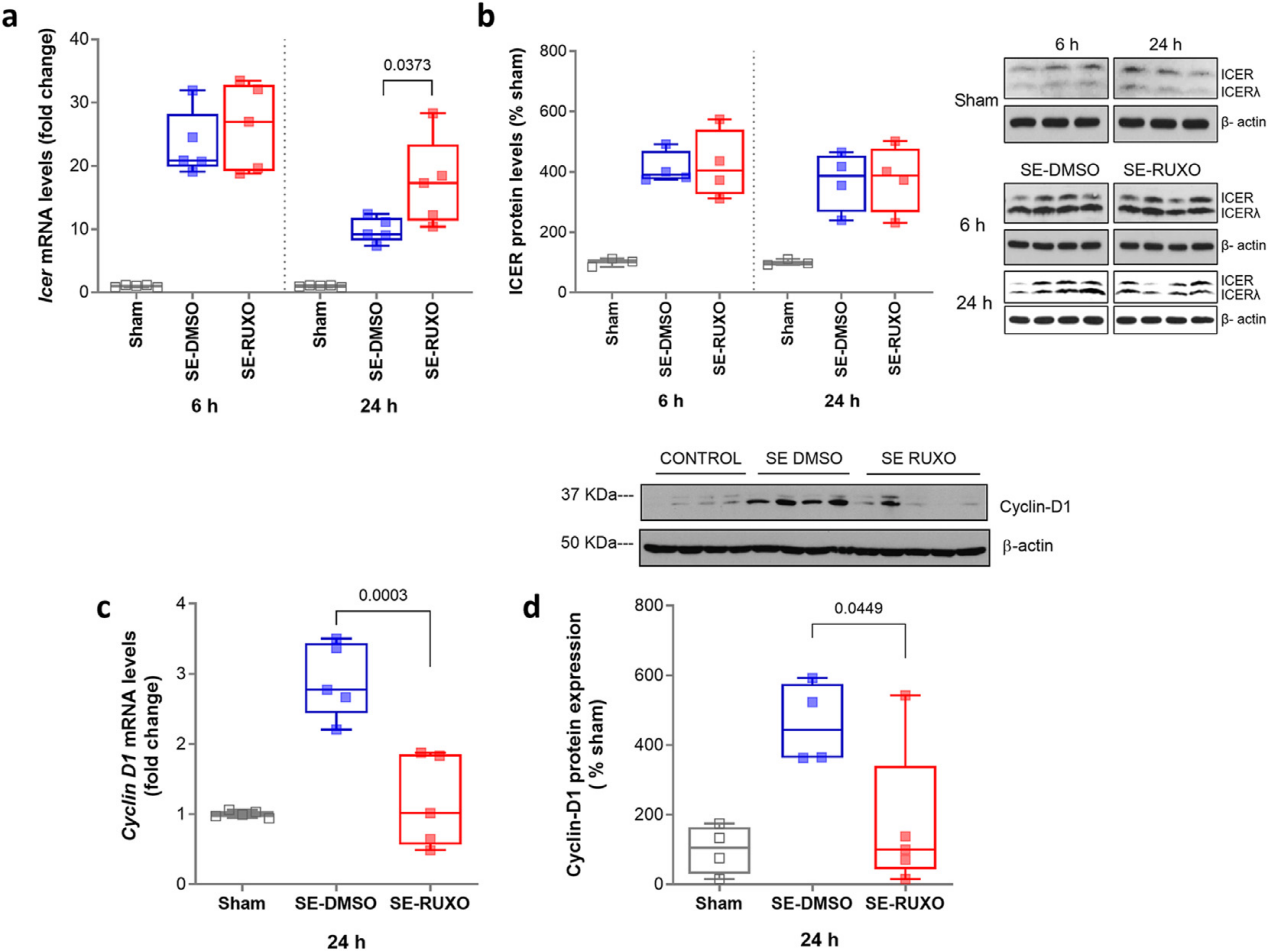
**mRNA and protein expression of downstream targets of the JAK/STAT pathway, ICER and Cyclin D1.** (**a**) Quantification of RT-PCR analysis of ICER mRNA levels in DG of Sham, DMSO and RUXO (n ​= ​5 for all groups) at 6 and 24h after SE. SE caused a statistically significant increase in *Icer* mRNA levels compared to Sham animals, not reversed by RUXO administration. Expression of mRNA was normalized to cyclophilin and expressed as fold change of controls. (**b**) Total ICER protein expression at 6 ​h and 24 ​h in DG of Sham (n ​= ​3) and SE rats (n ​= ​4 for both DMSO and RUXO groups) was assessed by western blot, normalized by β-actin and expressed as % of Sham. (**c**) *Cyclin D1* mRNA levels were assessed in DG of Sham, DMSO and RUXO animals (n ​= ​5 per group) at 24 ​h. Data are shown as fold change of Sham. (**d**) Total Cyclin D1 protein levels were evaluated at 24 ​h in DG of Sham (n ​= ​4), DMSO (n ​= ​4) and RUXO (n ​= ​5) rats and normalized by β-actin. Results are displayed as box and whiskers (min-max range) and expressed as % of Sham. Statistical analysis was performed with either unpaired *t*-test (**a**–**c**) or Mann-Whitney (**d**).

The effect of RUXO treatment on gene and protein expression of two downstream effectors of STAT3, namely inducible cAMP early repressor (ICER) and Cyclin D [16,27,31], was evaluated in DG at 6 ​h and 24 ​h after SE onset, time points at which *Icer* gene expression is known to be increased [31]. Both DMSO- and RUXO-treated SE animals showed significantly increased *Icer* mRNA expression compared to Sham animals at 6 ​h (by respectively 22.4-fold and 25.1-fold, p ​< ​0.0001; Fig. 8a), with no significant differences recorded between the SE-DMSO and SE-RUXO groups. The observed increase of *Icer* mRNA in SE animals was sustained at 24 ​h post-SE with both groups still showing significantly elevated levels of *Icer* mRNA relative to Sham (8.8- and 16.3-fold respectively for DMSO- and RUXO-treated rats). However, unlike at the earlier time points, at 24 ​h post-SE a statistically significant increase in *Icer* mRNA was observed in SE-RUXO animals compared to SE-DMSO group (7.5-fold increase; Fig. 8a).

ICER protein quantification largely paralleled the gene expression results, with both SE groups showing ∼ 3-fold increase compared to Sham, both at 6 ​h and 24 ​h (Fig. 8b). No difference was detected between DMSO and RUXO groups at either time point.

We next investigated mRNA and protein levels of *Cyclin D1*, another downstream effector of STAT3, in DG at 24 ​h after SE onset, the earliest time point at which *Cyclin D1* expression has been shown to be upregulated as a results of SE [31]. At this time point, the DMSO group showed *Cyclin D1* mRNA and protein levels respectively of 2.9-fold and 4.6-fold (Fig. 8c, d) compared to Sham. Mimicking what was previously observed with WP1066 [31], in SE-RUXO-treated rats *Cyclin D1* levels were significantly lower both at mRNA and protein level compared to DMSO-SE rats (respectively 0.404- and 0.377-fold of SE-DMSO; Fig. 8c, d).

## Discussion

Studies from our and other groups have produced evidence that epileptogenic brain injuries, such as SE, TBI and stroke, activate the JAK/STAT pathway [[16], [17], [18], [19],^31^,^34^], and that pharmacological inhibition of this pathway improves vestibular motor function in a mouse model of TBI [34]. In agreement with these earlier findings, recent work on our *camk2a-cre/ERT2/Stat3*^*fl/fl*^ mice demonstrated that selective knock-out of *Stat3* in excitatory neurons led to reduction of IHKA-dependent seizure progression and hippocampal memory deficits [30]. These changes were accompanied by modulation of gene expression in major networks associated with response to brain injury, neuronal plasticity, and learning/memory, suggesting a putative role for neuronal STAT3 in brain inflammation.

In search for a pharmacological intervention that could target the epileptogenic processes underlying TLE, our prior findings in the PILO rat model of epilepsy indicated that administration of the STAT3 inhibitor WP1066 at the time of SE reduced the number of spontaneous seizures after the latent period [31]. In addition, WP1066 modulated the expression of genes involved on one hand in synaptic plasticity, neurogenesis, and axonal remodeling, and on the other hand in the epileptogenic process following brain injury. Notably, however, while pSTAT3 inhibition by WP1066 improved injury-induced vestibular motor dysfunction following experimental TBI, it did not enhance memory [34] nor did it reduce the development of post-traumatic epilepsy, suggesting that WP1066 could mediate some but not all aspects of recovery after brain injury.

In the current study, we sought to determine if peripheral administration of a newer JAK inhibitor, ruxolitinib (RUXO), produced a more effective and prolonged inhibition of STAT3 phosphorylation when administered 30 ​min post-SE, and if this would result in a greater reduction ─ or prevention─ of spontaneous seizures and/or improved cognitive function, translating to a more robust disease modification effect. Ruxolitinib has been developed and is currently FDA-approved for the treatment of high-risk myelofibrosis, polycythemia vera, and steroid-refractory acute graft-versus-host disease [51]. Studies in mice have shown that RUXO was effective at crossing the blood–brain barrier (BBB), reduced brain astrogliosis associated with HIV encephalitis [52], and exerted a neuroprotective effect on TBI inhibiting the process of ferroptosis even at the low dose [53].

We found that in our Sham rats RUXO had an *in vivo* half-life of 75–90 ​min, and that 20 ​mg/kg RUXO, administered i.p. 30 min post-SE onset, resulted in mean brain concentrations of 600 ​nM at 1 ​h (=196 ​ng/g tissue, ∼34-fold higher than what observed at the same time point upon administration of 2 doses of 50 ​mg/kg WP1066 [31]) and 362.5 ​nM at 3 ​h, another confirmation that this drug crosses the BBB with higher efficacy, and clearance is considerably slower than WP1066 [31]. When animals were administered a second dose of 10 ​mg/kg of ruxolitinib 3 ​h post-SE, mean brain concentrations of 417.8 ​nM were maintained at 6 ​h. These concentrations in brain were in 10-fold excess of the *in vitro* IC_50_ for pSTAT3 inhibition (5.73 ​nM) and resulted in nearly complete inhibition of STAT3 phosphorylation in hippocampus at 1 ​h after SE onset, and sustained pSTAT3 reduction through 6 ​h after SE onset.

RUXO treatment beginning at 30 ​min after SE onset resulted in an overall reduction in total seizure load assessed in the 4 weeks of recording to ∼50 ​% of that seen in vehicle-treated epileptic rats. Furthermore, RUXO produced a profound and significant reduction in seizure duration beginning at week 2 of recording, sustained and amplified throughout the following 2 weeks. Interestingly, this response was relatively less than the effect of WP1066 (which produced a sustained and significant reduction - ∼85 ​% - of the frequency of spontaneous seizures 5–14 days post-SE [31]), despite the more extensive and prolonged inhibition of STAT3 phosphorylation that RUXO produced.

Behaviorally, RUXO-treated SE rats did not show differences in locomotor ability compared to DMSO-treated ones. Similarly, the degree of thigmotaxis (measure of anxiogenic behavior [54]), was not different between RUXO- and vehicle-treated rats. Conversely, RUXO seemed to moderately improve spatial working memory 4 weeks post-SE, in agreement with our most recent findings of improved motor learning and memory in the IHKA mouse model of TLE upon selective genetic inhibition of STAT3 activity in excitatory neurons [30]. Our behavioral findings partly agree with a recent study which showed that RUXO-prenatally treated adult mice displayed increased explorative behavior within an open field and improved spatial learning and long-term memory retention, not accompanied by changes in motor coordination, locomotor function, and recognition memory [55].

In terms of brain microstructural changes, in agreement with what was observed in our IHKA mouse model, in the current PILO rat model, mossy fiber sprouting, a hallmark of mesial TLE in humans and thought to contribute to epileptogenesis by mediating generation/propagation of spontaneous seizures [56], was evident 4 weeks after SE induction. As observed upon nSTAT3 KO [30], RUXO did not impact hippocampal lower or upper DG MFS, providing further confirmation that this phenomenon is not downstream of the JAK/STAT pathway. IHC staining with FJC displayed neuronal degeneration in hippocampal subregions (DG, CA1 and CA3) of SE rats, not rescued by RUXO treatment, similar to what was observed upon STAT3 KO in the IHKA mouse model [30].

It was unexpected that despite the increased degree and duration of the inhibitory effect of RUXO on STAT3 phosphorylation in hippocampus, which was substantially greater than what was previously preported with WP1066 [31], ruxolitinib had a less robust effect on SRS than did WP1066. We have previously shown that activation of the JAK/STAT pathway after SE causes increased transcription of *Icer* and subsequent down-regulation of α1-containing GABA_A_ receptors [16], a downregulation also observed in epilepsy patients [57]. Interestingly, in primary neocortical neurons *in vitro,* low concentrations of RUXO (100 ​nM; in the range of brain levels attained *in vivo* in the current study) inhibited STAT3 phosphorylation at Tyr 705 but did not lead to changes in the brain-derived neurotrophic factor (BDNF)-induced regulation of *Icer* expression. Conversely, higher RUXO doses (10 ​μM; 100-fold higher than *in vivo* levels attained in current study) successfully inhibited both pSTAT3 and *Icer*, suggesting that inhibition of STAT3 phosphorylation may be necessary, but not sufficient to inhibit *Icer*, and that BDNF-mediated JAK/STAT signaling may be more complex than originally described [27].

Supporting evidence for this concept comes from the finding that in the PILO rat model, mRNA levels of the STAT3 downstream target gene *Cyclin D1* and *Icer* were increased at 6 and 24 ​h after SE (current study and [31]), and administration of WP1066 rescued *Cyclin D1* mRNA levels 24 ​h after treatment, while *Icer* transcription levels were unchanged at the same time point [31]. In the current study, RUXO administration produced almost identical effects, leading to a substantial and significant reduction of *Cyclin D1* mRNA and protein levels both at 6 and 24 ​h, not accompanied by changes in *Icer* mRNA or protein levels at either time point. This apparent discrepancy can be explained by the existence of two different modes of JAK/STAT signaling. The canonical mechanism involves the JAK2-mediated phosphorylation of a critical STAT3 C-terminal tyrosine around the 700-amino-acid position (Y705) resulting in the translocation of STAT3 dimers into the nucleus where they function as transcriptional activators [58]. The transcriptional regulation of the *Cyclin D1* gene (among many others) has been shown to fall within the boundaries of this canonical pathway, via interaction with phospho-STAT3 [59], which seems to be confirmed by our findings. On the other hand, the non-canonical mechanism involves JAK2-mediated gene regulation via an interaction with the heterochromatic protein 1 (HP1 [60]) and a pool of unphosphorylated STATs that accumulate in the nucleus in response to IL-6 and activate transcription via NFkappaB [61]. We theorize that *Icer* transcriptional regulation may be modulated by both pathways. In agreement with this, ICER has been shown to be predominantly induced by IL-6 via STAT3 signaling in Th17 memory cells [62]. We have recently shown that in primary neocortical neurons, a number of modulators of synaptic plasticity, neurogenesis, and axonal remodeling - many of which are related to epilepsy-, are regulated through this non-canonical pathway [27]. In the same study, we produced evidence that similarly to JAK2, STAT3 can also associate with HP1 [27]. In these studies, *Gabra1* regulation via ICER is independent of STAT3 phosphorylation, suggesting that neuronal signaling through both the canonical and non-canonical pathways may be relevant to epilepsy, and potentially to learning and memory.

Our current findings suggest that RUXO may exert its seizure- and behavior-dependent effects by acting on the canonical pathway in neurons, and that ICER expression could be an effective biomarker of non-canonical JAK/STAT-mediated signaling that may also need to be targeted via an alternative therapeutic strategy to obtain maximal disease modification from JAK/STAT inhibitors.

Our most recent pharmacological studies targeting the JAK/STAT pathway provide confirming evidence that inhibitors of these pathways (both canonical and non-canonical) are a promising potential therapeutic approach to inhibit primary epileptogenesis after brain injury and to reduce severity of acquired epilepsy. As in the case of our previous studies with WP1066, in the current study we administered RUXO peripherally following SE onset, making it a potentially practical and therapeutically relevant approach for clinical practice. Compared to WP1066, RUXO's greater stability and BBB permeability resulted in higher efficacy as an inhibitor of STAT3 phosphorylation, and translated into disease modifying effects including significantly shorter seizure duration and lower seizure frequency throughout the 4 weeks of EEG recording.

Although it had a disease-modifying effect, RUXO treatment did not prevent development of epilepsy when administered at the time of SE. As our recent studies in the IHKA model demonstrate that enduring genetic inhibition of STAT3 activity in excitatory neurons [30] prevents epilepsy progression, future studies should also investigate whether pharmacological inhibition of STAT3 activity with RUXO after the onset of epilepsy provides a similar benefit. Furthermore, as discussed in the case of WP1066, kinase inhibitors are highly nonspecific. As such, we cannot exclude the possibility that the effect of RUXO on spontaneous seizure severity and/or frequency may be reduced by off-target effects on other pro-epileptogenic signaling pathways. In agreement with this, epileptic seizures have been reported concomitantly with the administration of RUXO when employed as pharmacological therapy in a case of Polycythemia vera [63]. Furthermore, RUXO has not been optimized for use in neurological disorders in humans, and a myriad of direct and off-target side effects on CNS have been attributed to RUXO and other JAK/STAT pathway inhibitors spanning from dizziness, peripheral neuropathy, ataxia, speech dysfunction, amnesia, to Wernicke encephalopathy [64], which may limit the usefulness of current STAT3 inhibitory molecules. As discussed above, we also believe RUXO may not target the non-canonical pathway of JAK/STAT signaling in neurons given the lack of inhibition of ICER induction at the tested dose. Ongoing studies in our laboratories aim to determine how this pathway specifically regulates the expression of so many ion channels and receptors in order to more effectively and specifically target it as a new therapeutic strategy for epilepsy intervention [27].

Finally, our study should be considered in light of the following limitations: 1) Similar to what was observed in the case of WP1066, the high dose of RUXO required to obtain a CNS effect after peripheral administration *in vivo* vs. effective concentration *in vitro* suggests the need for optimization of some pharmacodynamic/pharmacokinetic conditions for this drug to be employed for CNS clinical indications, especially in light of its potential adverse side effects; 2) A considerable variability has been recorded for weekly seizure frequency data, suggesting variable PILO- and RUXO-dependent effects resulting in the identification (and exclusion from statistical analysis) of a number of outliers (n ​= ​4 for DMSO-treated and 5 for RUXO-treated), which decreased the size of our groups for analysis; 3) Our behavioral data suffer from low sample size due to the exclusion of 5 animals (2 DMSO-treated and 3 RUXO-treated) as a result of complete lack of movement or seizing during testing sessions. As a result, our statistical analysis may be underpowered and significant differences recorded between the RUXO and DMSO groups during Y maze testing require confirmation with bigger sample size; and 4) Spatial memory was not evaluated in our previous study utilizing WP1066 in the pilocarpine model in rats, so a comparison between effects of RUXO vs. WP1066 for this parameter in the PILO model could not be presented.

## Authors Contributions

**AC:** Investigation; Formal Analysis; Writing - Review & Editing. **EN:** Formal Analysis; Visualization; Validation; Writing - Review & Editing. **KH:** Investigation; Data Curation; Formal Analysis; Validation. **JC:** Investigation; Data Curation; Formal Analysis. **YCDA:** Investigation; Data Curation; Formal Analysis; Validation; Writing - Review & Editing. **DS:** Investigation; Data Curation; Formal Analysis**. NB:** Investigation; Data Curation; Formal Analysis. **VK:** Data Curation; Formal Analysis. **MW:** Data Curation; Formal Analysis. **SR:** Conceptualization; Methodology; Supervision; Writing - Original Draft; Writing - Review & Editing; Funding acquisition; Resources**. ABK**: Conceptualization; Methodology; Supervision; Writing - Original Draft; Writing - Review & Editing; Funding acquisition; Resources.

## Data availability

Data will be made available on request.

## Declaration of competing interest

The authors declare the following financial interests/personal relationships which may be considered as potential competing interests: Amy Brooks-Kayal reports financial support was provided by National Institutes of Health. Shelley J. Russek reports financial support was provided by National Institutes of Health. Amy Brooks-Kayal reports financial support was provided by Citizens United for Research in Epilepsy. Shelley J. Russek reports financial support was provided by Citizens United for Research in Epilepsy. Michael F. Wempe reports financial support was provided by Citizens United for Research in Epilepsy. If there are other authors, they declare that they have no known competing financial interests or personal relationships that could have appeared to influence the work reported in this paper.
