# Supplementary material for: Ruxolitinib-dependent reduction of seizure load and duration is accompanied by spatial memory improvement in the rat pilocarpine model of temporal lobe epilepsy
Source: Neurotherapeutics. 2024 Dec 5;22(2):e00506. doi: 10.1016/j.neurot.2024.e00506 (PMC12014301; doi:10.1016/j.neurot.2024.e00506)
Supplement: Multimedia component 1 [file mmc1.docx]

**Supplementary Information**

**Ruxolitinib-dependent reduction of seizure load and duration is accompanied by spatial memory improvement in the rat pilocarpine model of temporal lobe epilepsy**

Andrew Carrel*, Eleonora Napoli†, Kathryn Hixson^‡^, Jessica Carlsen^§^, Yasmin Cruz Del Angel†, Dana Strode^§^, Nicolas Busquet^¶^, Vijay Kumar^||^, Michael F. Wempe^||#^, Shelley J. Russek^‡, **,1^, and Amy R. Brooks-Kayal†^,1,a^

*Department of Pediatrics, University of Colorado School of Medicine, Aurora, CO, USA

†Department of Neurology, University of California Davis School of Medicine, Sacramento, CA, USA

^‡^Graduate Program for Neuroscience, Center for Systems Neuroscience, Boston University, Boston, MA, USA

^§^Department of Pediatrics, University of Colorado School of Medicine, Aurora, CO, USA

^¶^Department of Neurology, University of Colorado School of Medicine, Aurora, CO, USA

^||^Department of Pharmaceutical Sciences, Skaggs School of Pharmacy and Pharmaceutical Sciences, University of Colorado Anschutz, Aurora, CO, USA

^#^ Department of Chemistry, Kentucky State University, Frankfort, KY, USA

**Department of Pharmacology and Experimental Therapeutics, Boston University School of Medicine, Boston, MA, USA

^1^Authors contributed equally


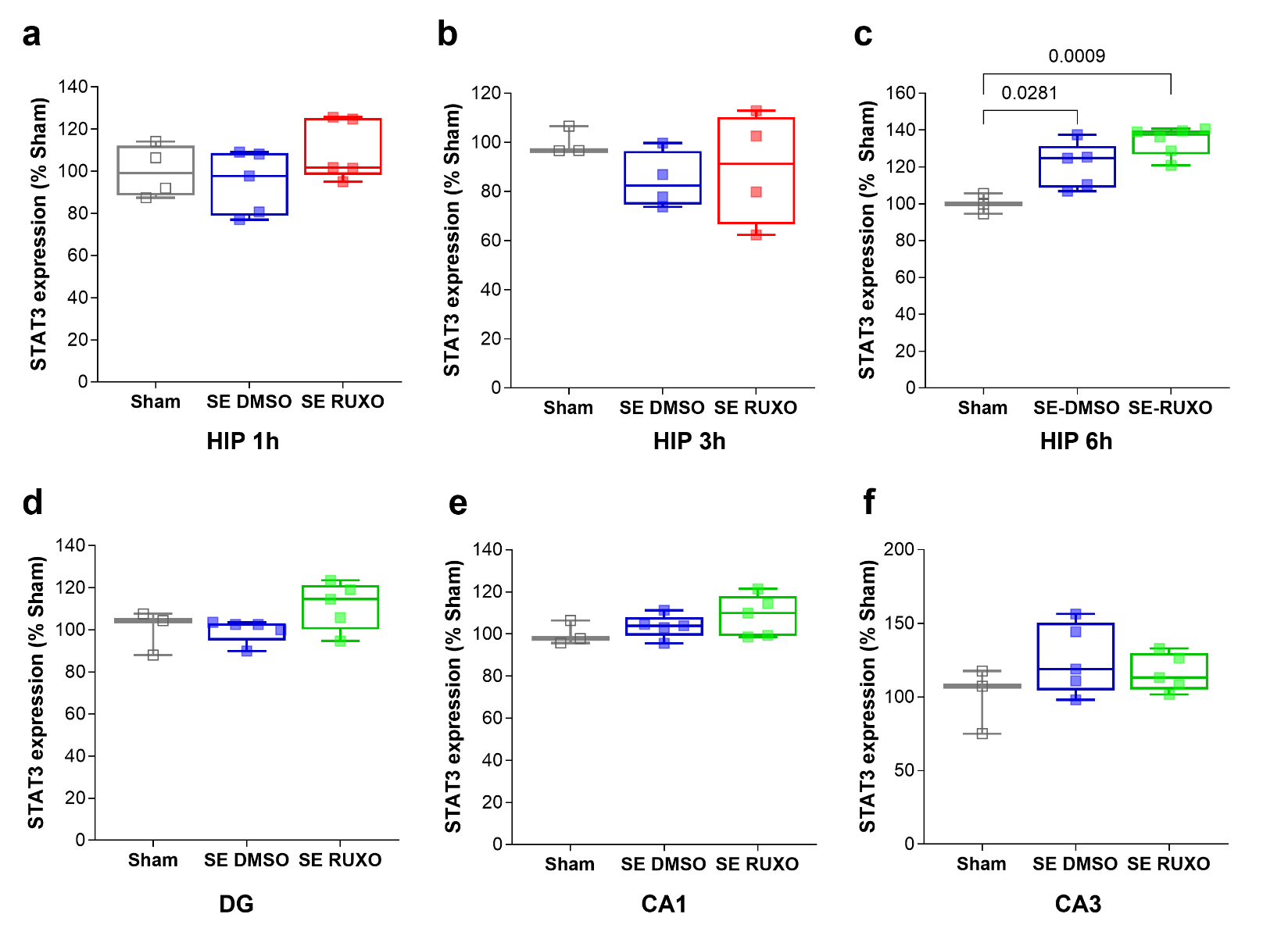


**Supplementary Figure 1 Total STAT3 expression in whole hippocampus and selected subregions of Sham and SE animals**

STAT3 expression levels of Sham (n = 4), DMSO (n = 5) and RUXO (n = 5) rats were normalized by β-actin (see Fig. 3 for representative immunoblot images), expressed as % of Sham and shown as mean ± SEM. STAT3 levels were measured in total hippocampus at 1, 3 and 6 h post-SE **(a-c)** or in the reported hippocampal subregions at 6 h post-SE **(d-f)**. Statistical analysis was performed by ANOVA followed by Fisher’s LSD post-test.


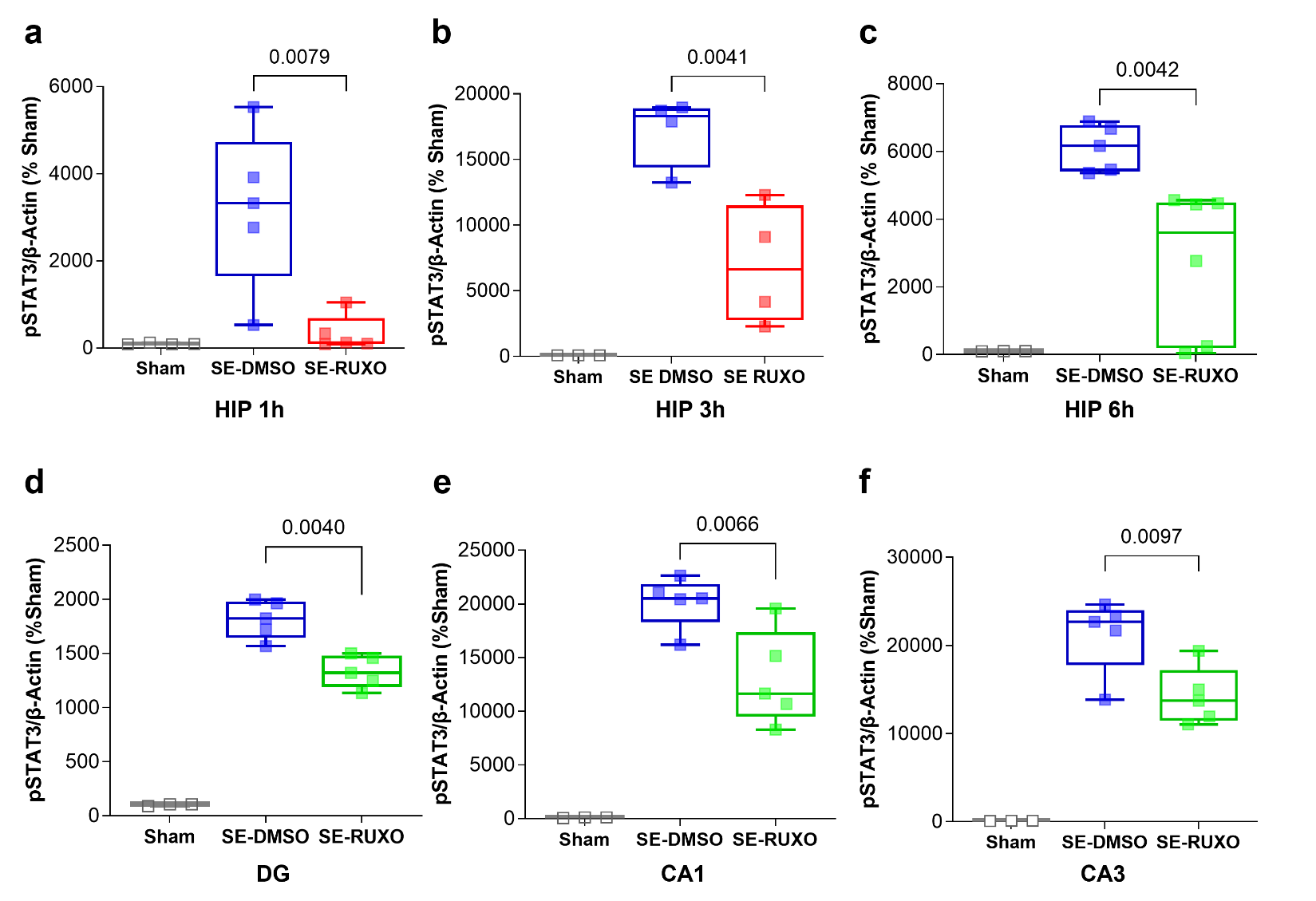


**Supplementary Figure 2 STAT3 phosphorylation levels in hippocampus and selected subregions upon normalization with β-actin**

Due to the recorded changes in total STAT3 levels in hippocampus at 6 h post-SE induction, normalization of phospho-STAT3 was carried out also by β-actin in total hippocampus at 1, 3 and 6 h (**a-c**), as well as in selected hippocampal subregions (DG, CA1 and CA3) 6 h after pilocarpine injection (**d-f**). Similar results were obtained with the two normalization methods (see Fig. 3 for phospho-/total STAT3 levels). Statistical analysis was performed by ANOVA followed by Fisher’s LSD post-test.


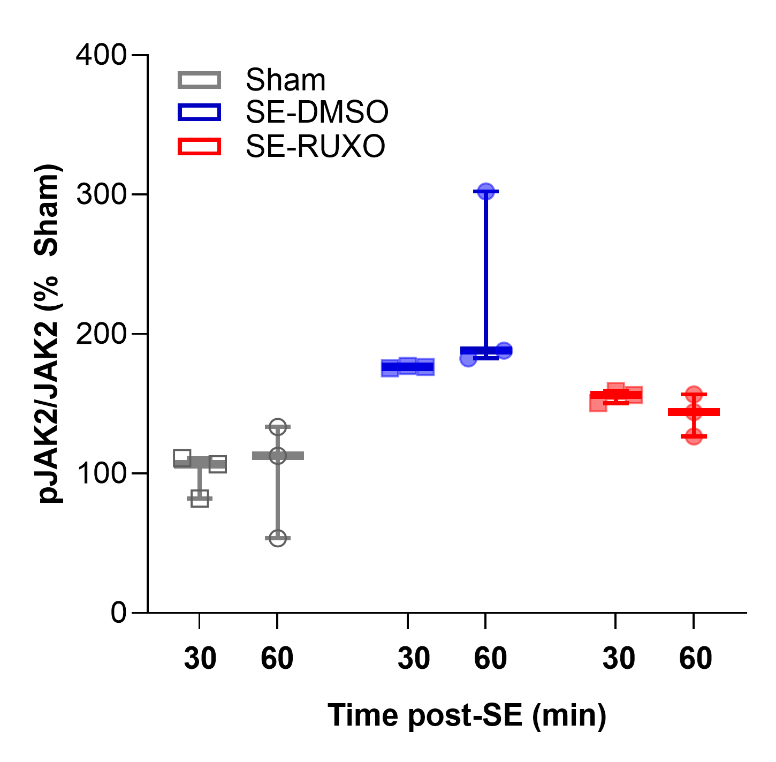


**Supplementary Figure 3 JAK2 phosphorylation levels in hippocampus at 30 and 60 minutes**

No differences were recorded in pJAK2 levels at 30- and 60-minutes post-SE. Given the low n (= 3 per time point per group), statistical analysis was run by Mann Whitney t-test comparing the two time points for each group.


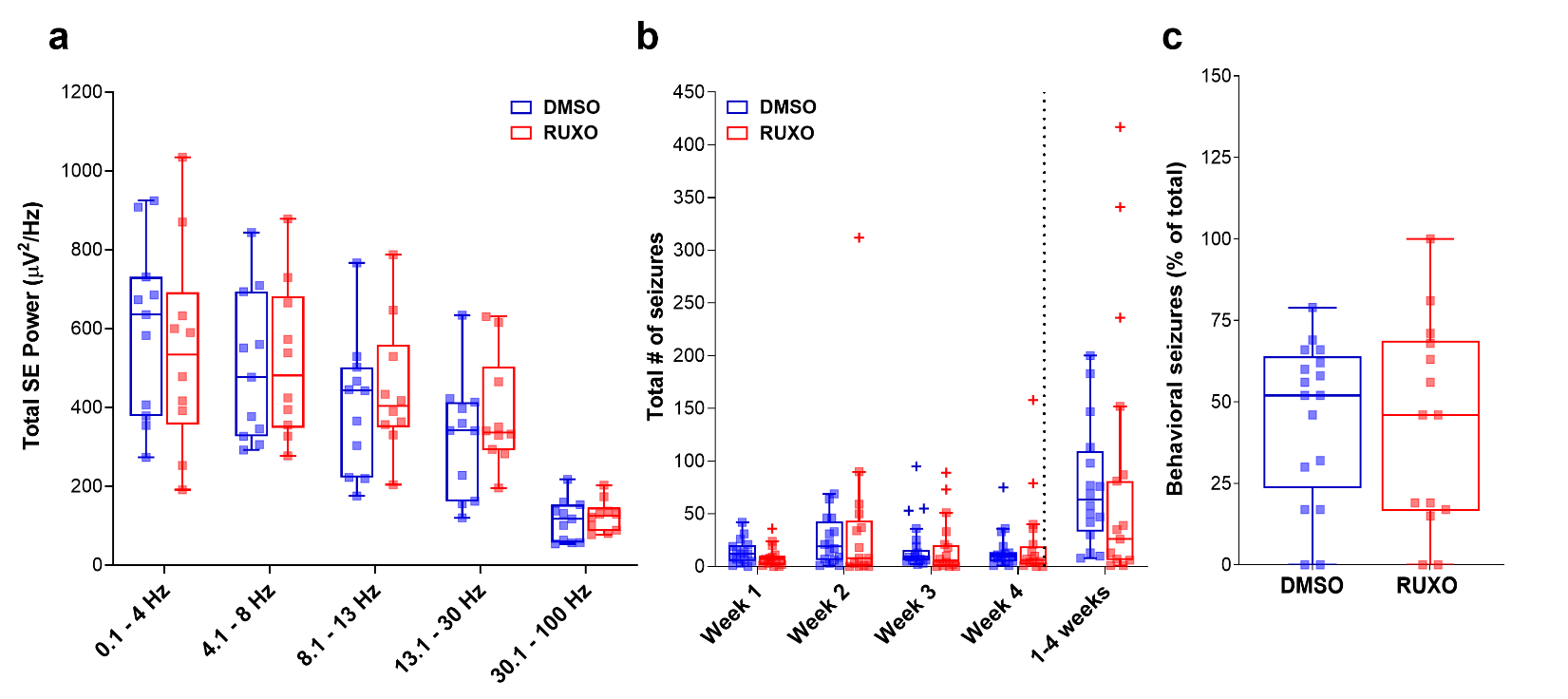


**Supplementary Figure 4 SE power and severity are not different between DMSO and RUXO animals**

Total SE power (0.1 – 100 Hz; **a**) was recorded in DMSO- and RUXO-treated animals for the duration of SE (defined as time from onset to 25% of maximum power). Thus, the total power metric incorporates both severity and duration of SE. No statistically significant difference was recorded for these parameters between groups, as indicated by comparable EEG integrated power at multiple bandwidths (x axis). Statistical analysis was performed with 2-way ANOVA followed by Šídák's multiple comparisons test. Seizure number per week (**b**) are shown including outliers (indicated by +) identified with the ROUT method (Q = 1%). Percentage of behavioral seizures (**c**) are shown as compared to total seizures (behavioral + electrographic). No difference was identified by unpaired Student’s t test (p = 0.8519).
